# A case with neurological abnormalities caused by *Rickettsia raoultii* in northwestern China

**DOI:** 10.1186/s12879-019-4414-4

**Published:** 2019-09-11

**Authors:** Zhihui Dong, Yicheng Yang, Qian Wang, Songsong Xie, Shanshan Zhao, Wenbo Tan, Wumei Yuan, Yuanzhi Wang

**Affiliations:** 1 School of Medicine, Shihezi University, Shihezi, 832002 Xinjiang Uygur Autonomous Region China; 2 Kaifeng Central Hospital, Henan, 475000 China; 3 The First Affiliated Hospital of Shihezi University Medical College, Shihezi, 832002 China

**Keywords:** *Rickettsia raoultii*, Neurological abnormalities, Northwestern China

## Abstract

**Background:**

The number of new rickettsial species are rapidly increasing, and increasing numbers of *Rickettsia raoultii* (*R*. *raoultii*) infection cases have been detected in humans. However, neurological abnormalities caused by *R. raoultii* are rarely reported, especially in northwestern China.

**Case presentation:**

A 36-year-old Kazakh shepherd with an attached tick on part temporalis, presented with right eyelid droop, lethargy, fever, headache, fever (38.0–41.0 °C) and erythematous rash. The examination of cerebrospinal fluid (CSF) showed cerebrospinal pressure of 200 mm H_2_O, leukocyte count of 300.0 × 10^6^/L, adenosine deaminase of 2.15 U/L, and total protein concentration of 0.93 g/L. The diagnosis of *R*. *raoultii* infection was confirmed by six genetic markers, and semi-quantified by enzyme-linked immunosorbent assay for rickettsial antigen. The patient gradually recovered after treatment with doxycycline and ceftriaxone. *R*. *raoultii* DNA was found both in a tick detached from this patient and in 0.18% (2/1107) of blood samples collected from local shepherds.

**Conclusions:**

This is the first reported case with neurological abnormalities caused by *R. raoultii* in northwestern China. It is vital to detect rickettsial agents both in blood and CSF for tick bite patients with neurological abnormalities. Public health workers and physicians should pay attention to neurological abnormalities caused by *Rickettsia*.

## Background

Rickettsial diseases are prevalent worldwide, although the prevalent organisms differ in different geographical regions [1]. *Rickettsia raoultii* (*R. raoultii*) were firstly found in *Dermacentor nuttallii* (*D. nuttallii*) ticks in 1999 [2]. Subsequently, it was detected in members of genus *Haemaphysalis*, *Rhipicephalus*, *Hyalomma* and *Amblyomma* ticks, especially in European and Asian countries [3–7]. In Xinjiang (northwestern China), *R*. *raoultii* was highly prevalent, and 26.35% (263/998) *Dermacentor* genus ticks were molecularly tested positive [8]. In addition, *R*. *raoultii* infections have also been increasingly detected in humans, and mainly distributed in Europe and Far East of Russia [9–15]. In contrast, only few human infection cases have been reported in China [16–18]. In 2017, twenty-six tick bite patients infected with *R*. *raoultii* were reported. Their clinical syndrome ranged from asymptomatic infection to severe illness. The nonspecific manifestations were common, and included fever (100%), malaise (95%), myalgia (58%), lymphadenopathy (53%) and nausea (42%). Only 5% of them had rash, and 16% had eschar [17].

Here we report a case with neurological abnormalities caused by *R. raoultii* infection, which was confirmed using two complementary methods, enzyme-linked immunosorbent assay (ELISA) and polymerase chain reaction (PCR), followed by multi-gene sequencing. Epidemiological and rickettsial surveillance were also conducted in Xinjiang.

## Case presentation

**Fig. 1:**
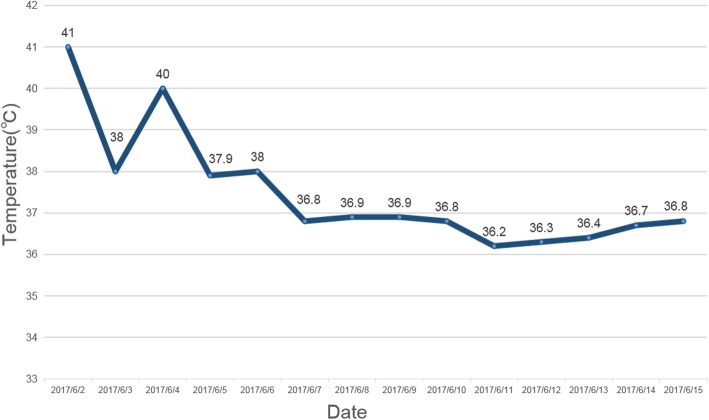
Change in patient’s body temperature

On June 3, 2017, a 36-year-old previously healthy Kazakh shepherd visited the First Affiliated Hospital of Xinjiang Medical University in Urumqi. He kept a tick detached from left part temporalis, which was identified as a female adult *D. marginatus* by an entomologist and further confirmed based on two tick-specific genetic markers [mitochondrial 16S ribosomal DNA (*16S rDNA*) and cytochrome c oxidase subunit I (*COI*)] according to previous reports [19]. The patient was initially asymptomatic, but 8 days later, his body temperature fluctuated between 38.0–41.0 (Fig. 1), accompanied by headache, malaise and anorexia. The patient gradually developed right eyelid droop, chest tightness, shortness of breath, lethargy and nausea accompanied by a vomiting 1 day before hospitalization. Cerebrospinal fluid (CSF) obtained through lumbar puncture examination showed cerebrospinal pressure of 200 mm H_2_O (normal range, 80–180 mm H_2_O), leukocyte count of 300.0 × 10^6^/L (normal range, 0–100 × 10^6^/L), adenosine deaminase of 2.15 U/L (normal range, 4–20 U/L), weakly positive Pandy test [20], and protein concentration of 0.93 g/L (normal range, 0.15–0.45 g/L). Transient leukocytosis developed after the onset and peaked on day 8 at 20.1 × 10^9^/L (normal range, 4–10 × 10^9^/L), with a neutrophilic leukocytosis. Other laboratory findings are shown in Table 1. A blood sample was collected and DNA was extracted using a TIANamp genomic DNA kit (Tiangen Biotechnique Inc., Beijing, China) according to the manufacturer’s instructions. Rickettsial DNA was primarily detected by 17 kilodalton antigen (*17*-*kDa*), which was *Rickettsia*-specific genetic marker [19]. The patient was treated with nasogastric or oral doxycycline 100 mg/day and Intravenous (IV) ceftriaxone 2 g/day for 10 days [12, 17]. Other symptomatic therapies like antipyretics, IV glycerol and fructose injection and IV mannitol were used as required. The patient was admitted to the intensive care unit for 6 days until fever disappeared and clinical manifestations alleviated. Repeated CSF test and routine blood test were normal. The patient has not experienced recurrence of fever or neurological symptoms after 6 months.

**Table 1:** Other laboratory findings from the patient

Laboratory findings			
Laboratory findings		results	normal range
Hemogram test	A white blood cell count	20.1 × 10^9^/L	4–10 × 10^9^/L
	Neutrophil count	18.03 × 10^9^/L	1.4–7 × 10^9^/L
	Lymphocyte count	0.82 × 10^9^/L	1.2–3.5 × 10^9^/L
	Hemoglobin level	122 g/L	110~ 160 g/L
	Platelet count	174 × 10^9^/L	100–300 × 10^9^/L
Blood biochemistry	Albumin	29.26umol/L	35-55umol/L
	Total bilirubin	7.35umol/L	2-28umol/L
	Glutamic-pyruvic transaminase (ALT)	74.72u/L	0-40u/L
	Glutamic-oxaloacetic transaminase (AST)	56.64u/L	0-40u/L
	Creatine kinase (CK)	330 IU/L	25-200 IU/L
	Potassium ion	3.29 mmol/L	3.5–5.5 mmol/L
	Uric aci (UA)	94umol/L	120-440umol/L
	Hypersensitive C-reactive protein	63.401 mg/L	0-6 mg/L
	Interleukin 6	41.910Pg/mL	<7Pg/mL
	Erythrocyte sedimentation Rate (ESR)	54.00 mm/h	0-15 mm/h
	ɑ1-acidic glycoprotein	1.56 g/L	0.51–1.17 g/L
	Transferrin (TRF)	1.43 g/L	2.02–3.36 g/L
	D-dimer	789.0 ng/ml	< 280 ng/ml
	Free thyroxine	10.66 pmol/L	12-22 pmol/L
	Free triiodothyronine	2.66 pmol/L	3.1–6.8 pmol/L
Routine urine	Urine protein	Positive(+ 2)	negative
	Urine acetone bodies	Positive(+ 2)	negative

**Fig. 2:**
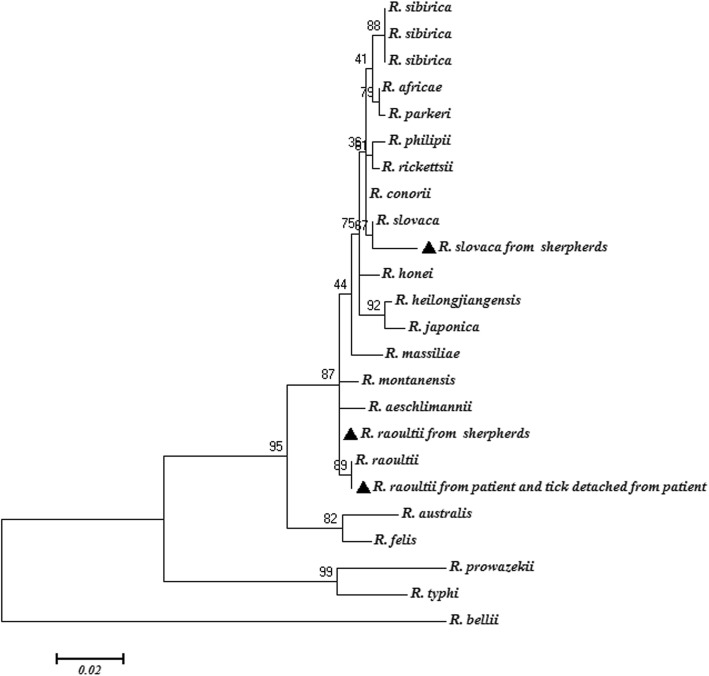
Phylogenetic tree of *17-kDa*-*glt*A-*sca*1-*sca*4-*omp*A-*omp*B concatenated sequences of *R. raoultii* in blood from patient, tick detached from patient and shepherds (▲). The target nucleotide sequences were compared to sequences that were available in public databases using BLAST (http://blast.ncbi.nlm.nih.gov/Blast.cgi). The tree was constructed on the basis of maximum-likelihood (ML; 1000 bootstrap replicates) of concatenated sequence data of six genes (*17-kDa*-*glt*A-*sca*1-*sca*4-*omp*A-*omp*B) using Molecular Evolutionary Genetics Analysis (MEGA, version 7.0; http://www.megasoftware.net/). The sequences of *R*. *bellii* were used as the outgroup

In order to investigate the presence of tick-borne viruses, RNA was extracted using an UItrapure RNA kit (CWBIO, Jiangsu Province, China) and complementary DNA was synthesized with random hexamers, using the Revert Aid First Strand cDNA synthesis kit (TRANSGEN BIOTECH, Beijing, China) for molecular detection of forest encephalitis virus, severe fever and thrombocytopenia syndrome virus, Crimean-Congo hemorrhagic fever virus and *Powassan* virus. Meanwhile, the other major tick-borne bacterial pathogens, such as *Anaplasma phagocytophilum*, *Ehrlichia chaffeensis*, *Babesia* spp., *Francisella tularensis*, *Brucella* and *Borrelia burgorferi* sensu lato, was also detected. All these pathogens were tested negative by PCR. To further determine the *Rickettsia* species, *Rickettsia* was molecularly detected by other five *Rickettsia*-specific genetic markers [surface cell antigen 4 (*sca*4); citrate synthase (*glt*A); cell surface antigen 1 (*sca*1); outer membrane proteins A (*omp*A); outer membrane proteins B (*omp*B)] as previously described, and *R*. *raoultii* was identified by sequencing [19]. Simultaneously, *R*. *raoultii* was detected in the feeding tick detached from the patient’s part temporalis using two *Rickettsia*-specific genetic markers (*Sca*4 and *omp*A). BLASTn (http://blast.ncbi.nlm.nih.gov/Blast.cgi) analysis revealed that the six gene fragments of *R*. *raoultii* had nucleotide identity of 99.5–100% with the reference *R*. *raoultii* strain Khabarovsk genome (accession number: CP010969) (Fig. 2). *R*. *raoultii* detected in the feeding *D. marginatus* tick detached from the patient was identical to those amplified from the patient.

To determine rickettsial antigen content at different stages of treatment in the patient’s blood, heparinized blood samples obtained from the patient 1 and 7 days after admission were tested using human rickettsia ELISA kits according to the manufacturer’s instructions (Shanghai enzyme-linked immunization, Shanghai, China). Rickettsial antigen content gradually decreased after treatment (the results were 33.98 pg/ml after first day of hospitalization and 17.78 pg/ml after seventh day of antibiotic therapy, respectively).

To further investigate the prevalence of rickettsial infection in shepherds, 1107 blood samples were collected from the local human population in Manasi County, Xinjiang. DNA was extracted from anti-coagulated blood samples as described above. PCR was performed to amplify rickettsial *Sca*1 or *omp*A fragments, followed by sequencing [19]. Two *R*. *raoultii* and one *R*. *slovaca* DNAs were detected (Fig. 2). Fifteen nucleotide sequences have been deposited in GenBank [*17 kDa*: MG190332; *glt*A: MG190324; *sca*1: MG811838, MK562056, MK535095, MG190331; *sca*4: MG190326, MK721054; *omp*A: MG190325, MK721055-MK721057; *omp*B: MH036479; *16srDNA*: TMK813858; *COI*: TMK813859].

## Discussion and conclusions

It is well known that Q fever, spotted fever group (SFG) and typhus group rickettsial infection may cause central nervous system infection [21]. Among SFG *Rickettsia*, *R*. *rickettsii*, *R*. *conorii*, *R*. *helvetica*, Candidatus *R. tarasevichiae* and *R. japonica* have documented association with meningitis [21–24]. In China and Europe, 2 Patients infected with *R*. *raoultii* were reported to show meningeal syndrome, respectively [17, 25]. In this study, the patient showed right eyelid droop, lethargy, fever, headache, high cerebrospinal pressure and leukocytosis in CSF after bitten by *D. marginatus* tick. Our study has a limitation related to detection of *Rickettsia* DNA in blood but not in CSF sample. As for biochemical parameters in blood and CSF is unspecific to patients with *R*. *raoultii* infection, it is vital to detect rickettsial agents both in blood and CSF for tick bite patients with neurological abnormalities.

The previous recommended therapeutic regimen for rickettsiosis is administration of doxycycline or chloramphenicol [26]. In this study, the patient recovered after treatment with doxycycline & ceftriaxone. This finding indicates that doxycycline combined with ceftriaxone should be recommended when the tick bite patient shows neurological abnormalities caused by rickettsial infection.

In this study, two *R*. *raoultii* (0.18%, 2/1107) and one *R*. *slovaca* (0.09%, 1/1107) were molecularly detected in local shepherds. In addition, the prevalence of SFG *Rickettsia* in ticks was high in Xinjiang [8]. Therefore, public health workers and physicians need to be aware of the wide distribution and clinical complexity of rickettsial infection, especially higher risk for tick exposure.

## Data Availability

All data generated or analyzed during this study are included in this published article and its supplementary information files.

## Abbreviations

*16S rDNA*: mitochondrial 16S ribosomal DNA
*17-kDa*: 17 kilodalton antigen
*COI*: cytochrome c oxidase subunit I
CSF: Cerebrospinal fluid
ELISA: Enzyme-linked immunosorbent assay
*glt*A: citrate synthase
MEGA7: Molecular Evolutionary Genetics Analysis 7
*omp*A: outer membrane proteins A
*omp*B: outer membrane proteins B
PCR: polymerase chain reaction
*R*. *raoultii*: *Rickettsia raoultii*
*sca*1: cell surface antigen 1
*sca*4: surface cell antigen 4
SFG: spotted fever group
